# Ly6c as a New Marker of Mouse Blood Vessels: Qualitative and Quantitative Analyses on Intact and Ischemic Retinas

**DOI:** 10.3390/ijms23010019

**Published:** 2021-12-21

**Authors:** Marina Martínez-Carmona, Fernando Lucas-Ruiz, Alejandro Gallego-Ortega, Caridad Galindo-Romero, María Norte-Muñoz, María José González-Riquelme, Francisco J. Valiente-Soriano, Manuel Vidal-Sanz, Marta Agudo-Barriuso

**Affiliations:** 1 Grupo de Oftalmología Experimental, Instituto Murciano de Investigación Biosanitaria Virgen de la Arrixaca (IMIB-Arrixaca), 30120 Murcia, Spain; fernando.lucas@um.es (F.L.-R.); alejandrogallego@um.es (A.G.-O.); caridad.galindo@um.es (C.G.-R.); maria.norte@um.es (M.N.-M.); mariajosefa.gonzalez3@um.es (M.J.G.-R.); fjvaliente@um.es (F.J.V.-S.); manuel.vidal@um.es (M.V.-S.); 2 Departamento de Oftalmología, Facultad de Medicina, Universidad de Murcia, 30100 Murcia, Spain

**Keywords:** vessels, cell extravasation, vascular plexus, ischemia

## Abstract

Ly6c is an antigen commonly used to differentiate between classical and non-classical monocytes/macrophages. Here we show its potential as a marker of the mouse vasculature, particularly of the retinal vascular plexuses. Ly6c was immunodetected in several tissues of C57BL/6 mice using isolectin IB4 as the control of vasculature staining. In the retina, Ly6c expression was analyzed qualitatively and quantitatively in intact, ischemic, and contralateral retinas from 0 to 30 days after the insult. Ly6c expression was observed in all organs and tissues tested, with a brighter signal and more homogeneous staining than the IB4. In the retinas, Ly6c was well expressed, allowing a detailed study of their anatomy. The three retinal plexuses were morphologically different, and from the superficial to the deep one occupied 15 ± 2, 24 ± 7, and 38 ± 1.4 percent of the retinal surface, respectively. In the injured retinas, there was extravasation of the classically activated monocyte/macrophages (Ly6c^high^) and the formation of new vessels in the superficial plexus, increasing the area occupied by it to 25 ± 1%. In the contralateral retinas, the superficial plexus area decreased gradually, reaching significance at 30 days, and Ly6c expression progressively disappeared in the intermediate and deep plexuses. Although the role of Ly6c in vascular endothelial cell function is still not completely understood, we demonstrate here that Ly6c can be used as a new specific marker of the mouse vasculature and to assess, qualitatively and quantitatively, vascular changes in health and disease.

## 1. Introduction

One of the main fields of study within neuroscience deals with the relationship between the circulatory system and the central nervous system (CNS). The circulatory system is responsible for providing oxygen and nutrients to all cells in the body, including the brain, as well as for removing the CO_2_ discarded by them. Unlike what happens in other organs, the brain has almost zero capacity to store energy in the long term, and therefore requires a continuous flow of oxygen and nutrients. In fact, despite accounting for about 2% of the weight of an adult, the brain requires approximately 20% of the heart output [1]. Therefore, the physiological role of the circulatory system is crucial; proof of this is that most neurodegenerative diseases or brain illnesses are associated with blood supply disorders, such as brain stroke or edema [2,3].

The retina is metabolically very active and, like the brain, also requires a large supply of nutrients and oxygen. The blood supply of the mouse retina is established in two vascular systems: the choriocapillaris, which irrigates the pigment epithelium and the outer retina, and the central retinal artery, which irrigates the inner retina [4]. Once the central retinal artery enters the retina through the optic nerve, it divides into three plexuses: the superficial vascular plexus in the plane of the retinal ganglion cells (RGCs) and the nerve fiber layer, the intermediate vascular plexus above the inner nuclear layer, and the deep vascular plexus above the outer nuclear layer [4].

Retinal vasculature alterations can have important effects on the visual system. A correlation between inner vascular deficits and RGC loss observed after the induction of ocular hypertension has been shown [5]. Similar to cerebral infarction, retinal ischemia is a serious problem that can lead to a severe loss of vision and even blindness, especially among the elderly population [6]. Glaucoma is a group of eye diseases that damage the optic nerve, the health of which is vital for good eyesight [7]. Although there are several risk factors for glaucoma, such as age or genetics, the main one is a high intraocular pressure, which is believed to cause a vascular and RGC axonal impairment [8,9,10].

Understanding the physiology and anatomy of the well-organized retinal vascular system is essential to monitor visual health and detect abnormalities associated with pathologies, such as glaucoma or ischemia.

There are several markers and techniques to identify the mouse blood vessels, summarized in Table 1.

Among them, the isolectin IB4, which binds to the sugar residues of the glycocalyx present on the surface of the blood vessels, is one of the most used, especially in the retina [5,11,12,13,14,15,16]. It has been reported that it could be used as a premature indicator of endothelial regression, i.e., the pruning of superfluous connections by regression and the polarized migration of endothelial cells, since its lack of expression precedes that of other markers such as collagen IV [11]. However, in the CNS, IB4 also binds to activated microglia, impairing in some cases the visualization of the blood network [30]. Antibodies against ZO-1, cadherin, CD31, ICAM-2, and claudin-5 recognize the endothelial cell–cell junctions giving rise to a brighter signal where the cells attach to each other and a lower signal in the cytosol. The intravenous injection of fluorophore-coupled substances (e.g., dextran or gel-BSA) results in the fluorescent labelling of the whole space inside blood vessels and can be used to study vessel permeability [31]. Each marker has its advantages and disadvantages, but in mice none is as precise as the best marker for endothelial cells in rats, the rat endothelial cell antigen 1 (RECA1), which shows vascular endothelium cell-specificity both in vitro and in vivo [32].

Ly6c (lymphocyte antigen 6 complex, locus C1) is a monocyte/macrophage cell differentiation antigen commonly used to differentiate classical monocytes (Ly6c^high^) from non-classical ones (Ly6c^low^) [33,34]. Alliot et al., in 1998 [30], showed that Ly6c was expressed in the blood vessels of the mouse brain but not in microglial cells, and we wondered whether the same was true for the healthy and diseased retina. Thus, using Ly6c immunodetection and IB4 as the positive control, we have characterized in depth the three retinal vascular plexuses in healthy retinas and in both retinas after the unilateral induction of retinal ischemia using the acute ocular hypertension model (AOHT) [35,36,37].

## 2. Results

### 2.1. Determination of the Ly6c Value as a Vasculature Marker

**Figure 1:**
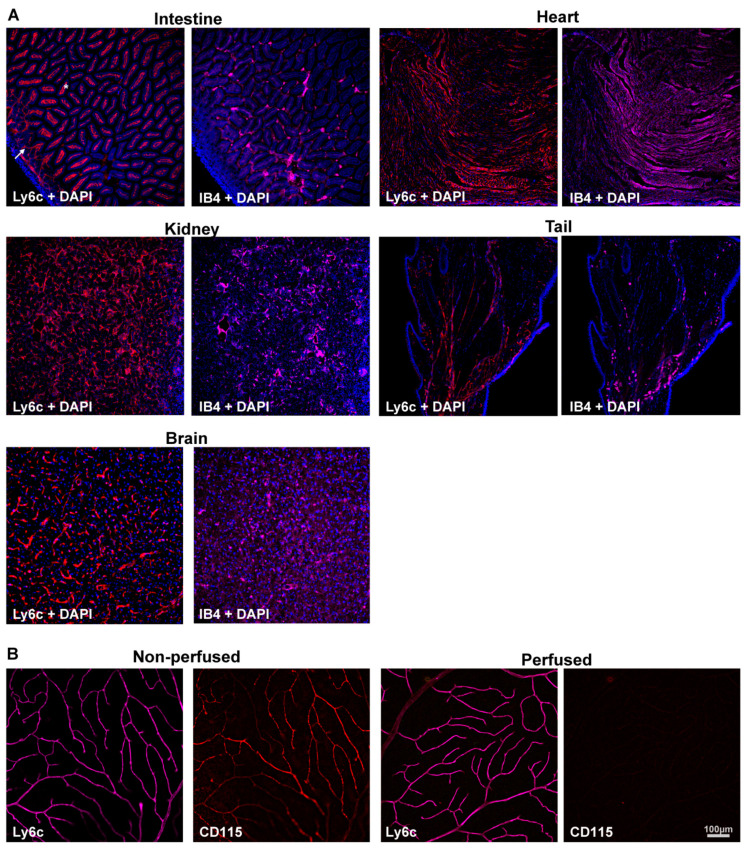
Ly6c is expressed in mouse blood vessels. (**A**) Ly6c (red) and IB4 (purple) detection in several tissues showing Ly6c signal in the brain, intestine, kidney, heart, and tail. The arrow points to blood vessels, and the asterisk to macrophages in the intestinal villi. Nuclei are counterstained with DAPI (blue). (**B**) Ly6c (purple) and CD115 (red) double immunodetection in non-perfused and perfused retinas. Scale bar is the same for all panels.

Ly6c and IB4, as the control of blood vessel staining, were detected in several mouse organs and tissues. Ly6c was expressed in the vasculature of all analyzed samples (Figure 1A), including the brain [30]. In the intestine, Ly6c is expressed in blood vessels, and by the macrophages lining the villi [38] (arrow and asterisks, Figure 1A). To ascertain whether Ly6c in the retina, labelled macrophages/monocytes associated with blood vessels, we immunodetected Ly6c and CD115 in perfused and non-perfused retinas. CD115 (also known as receptor for macrophage colony stimulating factor [39]) is expressed by monocytes, macrophages, osteoclasts, and some epithelial cells. The Ly6c and CD115 signal in non-perfused tissue was similar, evidencing the blood vessels. However, in the perfused retinas, while Ly6c expression in the vessels remained, the CD115 signal disappeared (Figure 1B), indicating that Ly6c does not label perivascular macrophages/monocytes.

### 2.2. Retinal Vasculature Visualized with Ly6c

**Figure 2:**
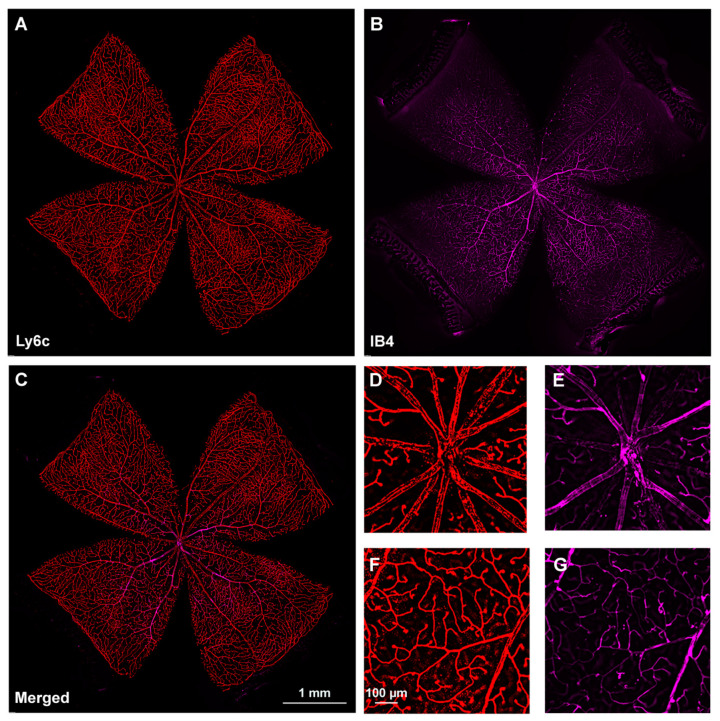
Comparison of Ly6c and IB4 vascular staining in the retina. (**A**–**C**) Photomontage of a representative intact retina showing Ly6C ((**A**), red), IB4 ((**B**), purple) and both signals (**C**) in the ganglion cell layer. (**D**–**G**) Magnifications from the central (**D**,**F**) and peripheral (**E**,**G**) retina are from the yellow squares in (**C**). Scale bar in (**C**) for (**A**–**C**) images, and in (**F**) for (**D**–**G**) panels.

Next, we investigated Ly6C expression in the retina, using IB4 as the positive control. Ly6c was strongly expressed in arteries, veins, and capillaries (Figure 2). In the central retina, Ly6c identified the 12 main superficial retinal vessels: 6 veins and 6 arteries arranged alternately while the IB4 signal was seen in only half of them, the arteries. In the retinal periphery, with thinner and smaller vessels, the IB4 signal was weaker and more difficult to visualize and image than the Ly6c signal (Figure 2, compare panels F and G).

**Figure 3:**
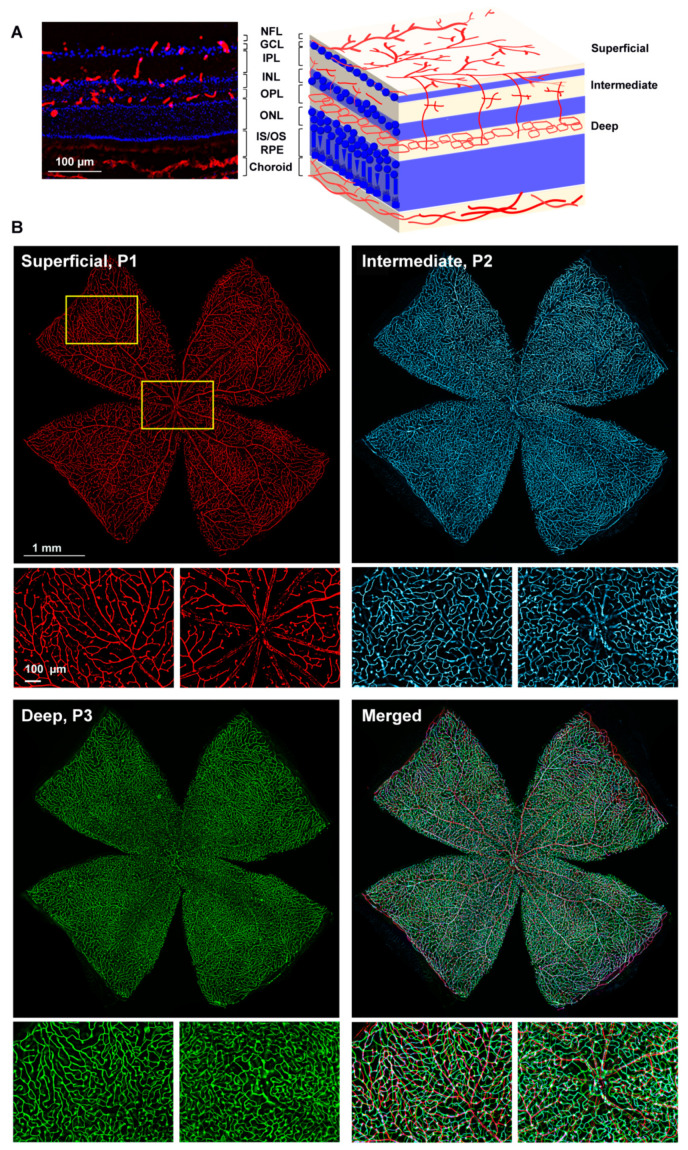
Retinal vascular plexuses. (**A**) Left: Ly6c immunodetection (red) in a retinal cross-section counterstained with DAPI (blue). Right: drawing depicting the retinal layers and the position of each vascular plexus. RGCL: retinal ganglion cell layer, IPL: inner plexiform layer, INL: inner nuclear layer, OPL: outer plexiform layer, ONL: outer nuclear layer. (**B**) Whole-mounted retina showing Ly6c staining in the three retinal vascular plexuses. Below each one there are magnifications from the two regions framed in yellow. 1 mm and 100 µm scale bars are for photomontages and magnifications, respectively.

**Figure 4:**
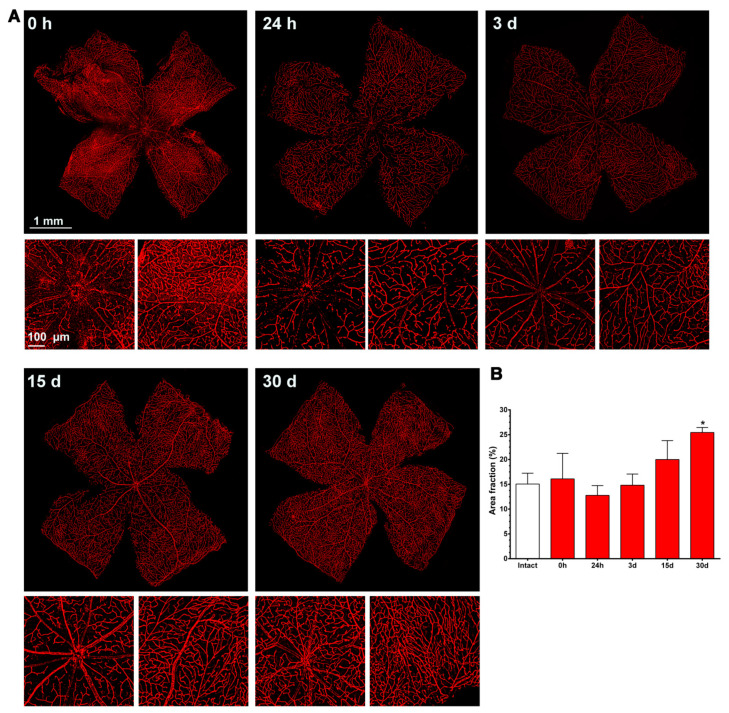
Changes in the retinal vasculature after transient ischemia. (**A**) Ly6c immunodetection showing the superficial plexus (P1) of ischemic retinas analyzed from 0 h (immediately after) to 30 days after the induction of the ischemia. Below each photomontage are shown magnifications of the optic nerve head or retinal periphery. (**B**) Bar graphs showing the area fraction of the retina covered by P1 ± standard deviation in intact and contralateral retinas. * Significant compared to intact retinas (*p* < 0.05, Kruskal–Wallis, Dunn’s multiple comparisons test). 1 mm and 100 µm scale bars are for photomontages and magnifications, respectively.

In the three retinal plexuses (P1, superficial; P2, intermediate; and P3, deep), the Ly6c signal was bright and clear (Figure 3A). Flat mounts show the spatial anatomy of each plexus distinctly (Figure 3B). The inner or superficial plexus, P1, is the more ramified, and the capillaries are capped with an engrossment, while P3 capillaries are saccular, with fewer free ends. P2 has an intermediate morphology. Finally, we measured the area of the retina occupied by the vasculature in each of the plexuses. In intact healthy retinas, 15 ± 2.2, 24 ± 7, and 31 ± 1.4% of the retinal area was occupied by P1, P2, and P3, respectively (graph in Figure 4).

### 2.3. Ly6c Identifies Vascular Changes in a Model of Retinal Ischemia

Once Ly6c expression in intact retinas was determined, we decided to validate its use in disease, using a model of retinal ischemia induced by increasing intra-ocular pressure by saline injection into the anterior chamber of the eye [36,37]. We only analyzed P1, because P2 and P3 could not be imaged due to retinal thinning (see below).

#### 2.3.1. Injured Retinas

**Figure 5:**
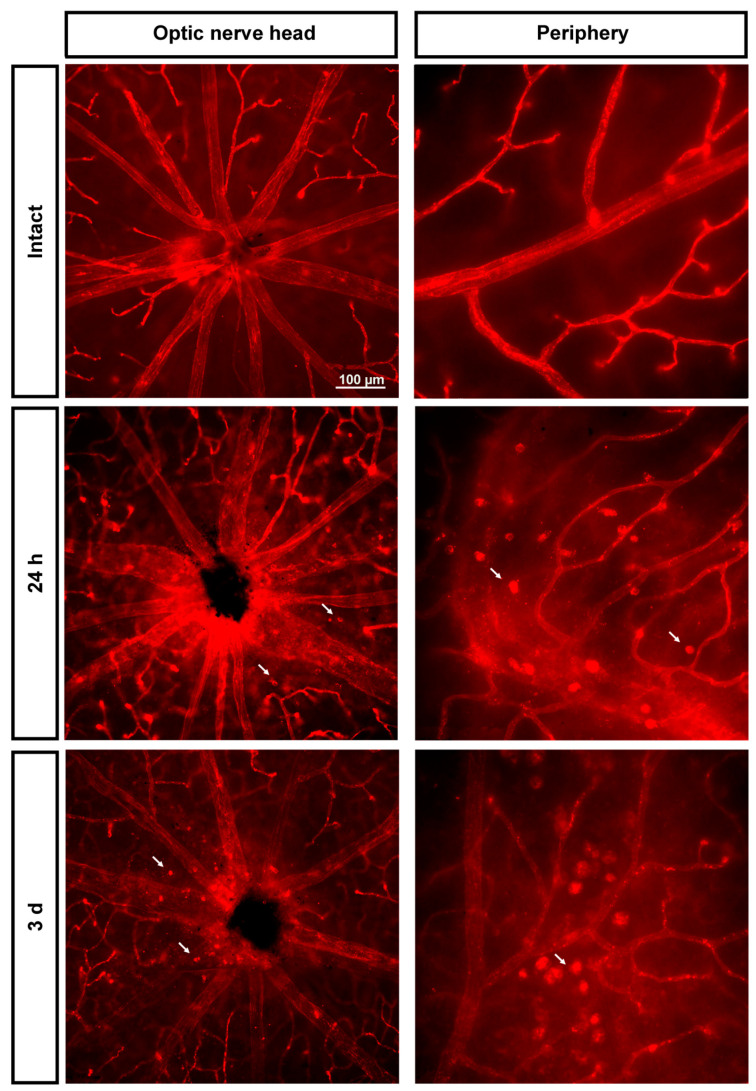
Extravasation of classically activated monocyte/macrophages early after the induction of transient ischemia. Ly6c expression in the superficial plexus around the optic nerve head and in the periphery of intact retinas and retinas analyzed 24 h and 3 days after the ischemia. Arrows point to extravasated classically activated monocytes/macrophages.

As shown in Figure 4, immediately after the ischemia retinas were quite damaged, but they recovered progressively. During the first 3 days, the expression of Ly6c around the optic nerve head decreased, and some major vessels showed a discontinuous Ly6c expression which could mean vessel rupture or down-regulation of this protein. At 24 h and 3 days, classically activated monocyte/macrophages (Ly6c^high^) were observed extravasated around the optic nerve head and in the periphery (Figure 5).

From 7 to 15 days there were no clear anatomical changes, although at 15 days there seemed to be more capillaries, with a tortuous morphology, in the periphery. At 30 days, the neo-vascularization was clearer and observed in the optic nerve head and the retinal periphery. Accordingly, the vascular fraction area of the retina increased significantly compared to that of the intact retinas (25 ± 1% vs. 15 ± 2%, graph in Figure 4).

**Figure 6:**
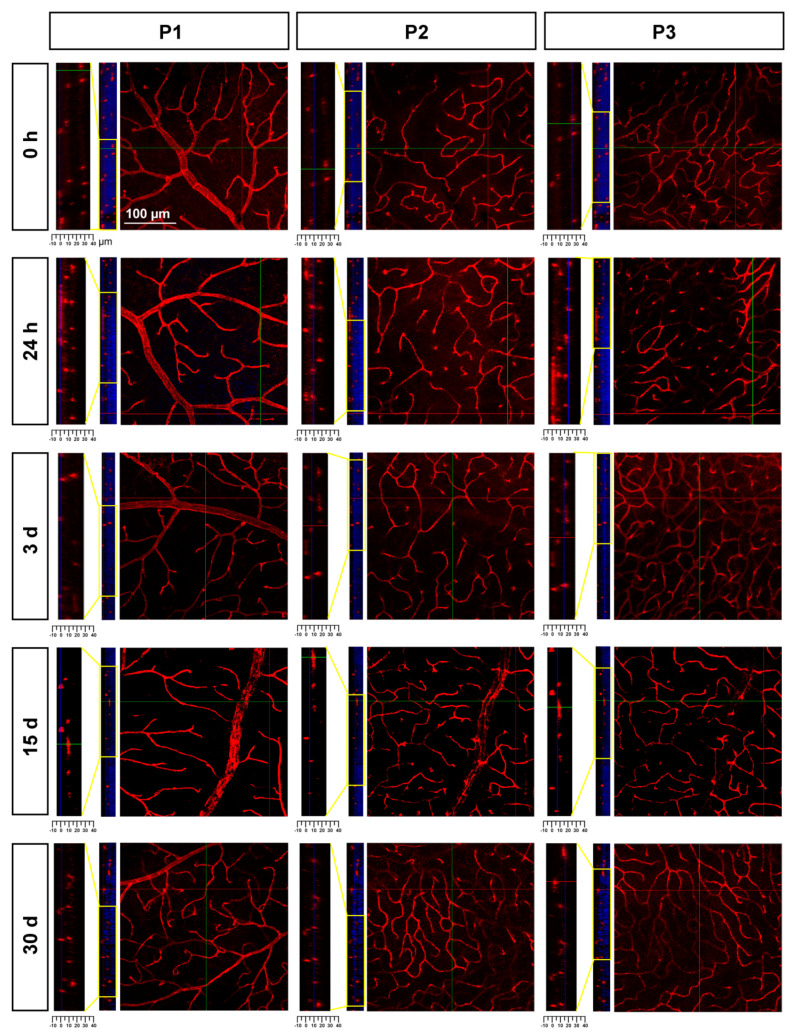
Retinal thinning after ischemia. Confocal images showing Ly6c signal in the three plexuses from 0 h to 30 days after the induction of the ischemia. To the left of each magnification is shown the z-stack spanning the retinal thickness (DAPI in blue) and a magnification of the z-stack (yellow square) where the ganglion cell layer is positioned a 0 µm in all images.

To study P2 and P3, we resorted to confocal imaging; this approach also allowed the changes in retinal thickness to be assessed. As seen in Figure 6, there was a progressive retinal thinning, as previously reported [37], and, as a result, P2 and P3 were practically indistinguishable at 30 days.

#### 2.3.2. Contralateral Retinas

**Figure 7:**
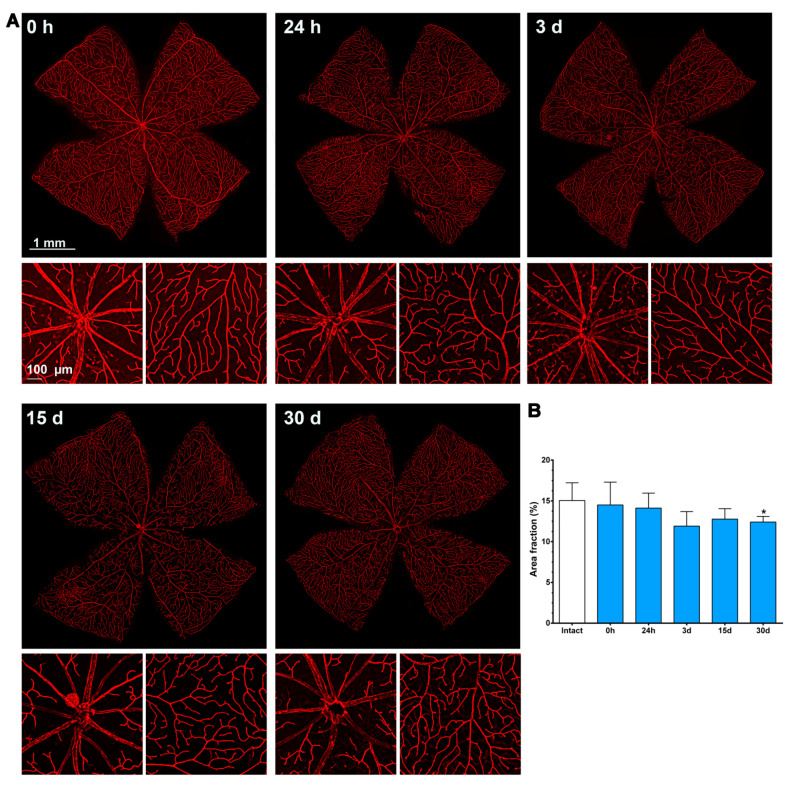
Changes in the retinal vasculature of the contralateral uninjured retinas. (**A**) Ly6c immunodetection showing the superficial plexus (P1) of contralateral retinas analyzed from 0 h (immediately after) to 30 days after the induction of the ischemia. Below each photomontage are shown magnifications of the optic nerve head or retinal periphery. (**B**) Bar graphs showing the area fraction of the retina covered by P1 ± standard deviation in intact and contralateral retinas. * Significant compared to intact retinas (*p* < 0.05, Kruskal–Wallis, Dunn’s multiple comparisons test). 1 mm and 100 µm scale bars are for photomontages and magnifications, respectively.

The retinal area fraction of the superficial plexus (P1) in the contralateral retinas diminished gradually, reaching significance compared to the intact retinas at 30 days (15 ± 2% vs. 12 ± 0.7, Figure 7).

**Figure 8:**
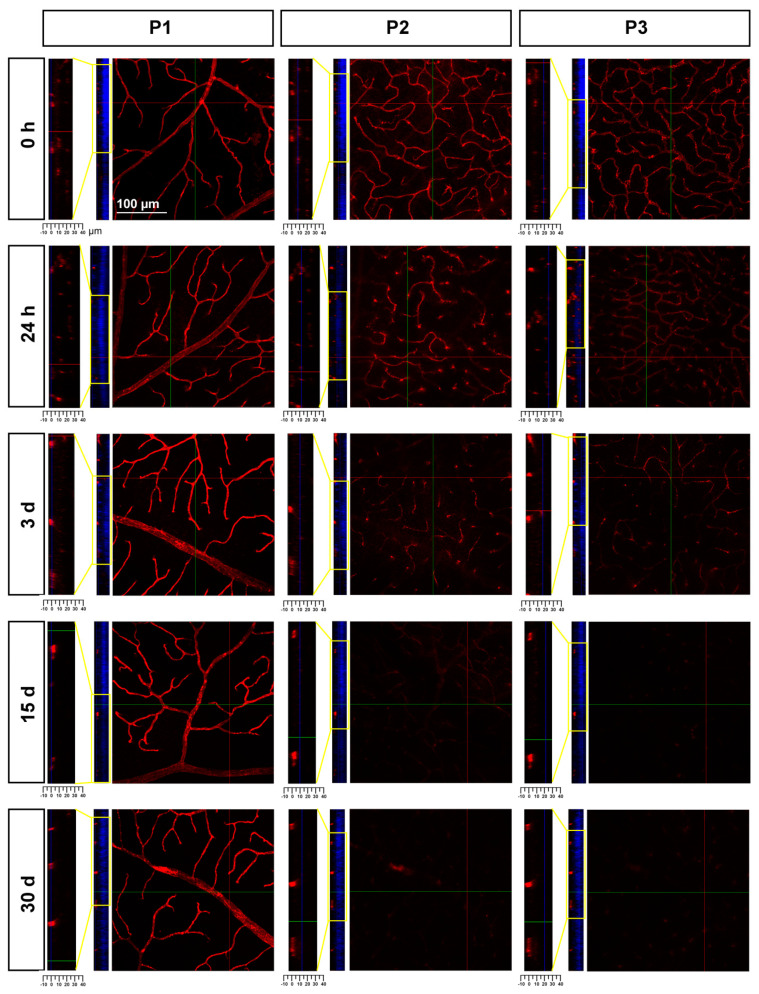
Loss of Ly6c expression in the intermediate and deep vascular plexuses of the contralateral uninjured retinas. Confocal images showing Ly6c signal in the three plexuses from 0 h to 30 days after the induction of the ischemia. To the left of each magnification is shown the z-stack spanning the retinal thickness (DAPI in blue) and a magnification of the z-stack (yellow square) where the ganglion cell layer is positioned a 0 µm in all images.

**Figure 9:**
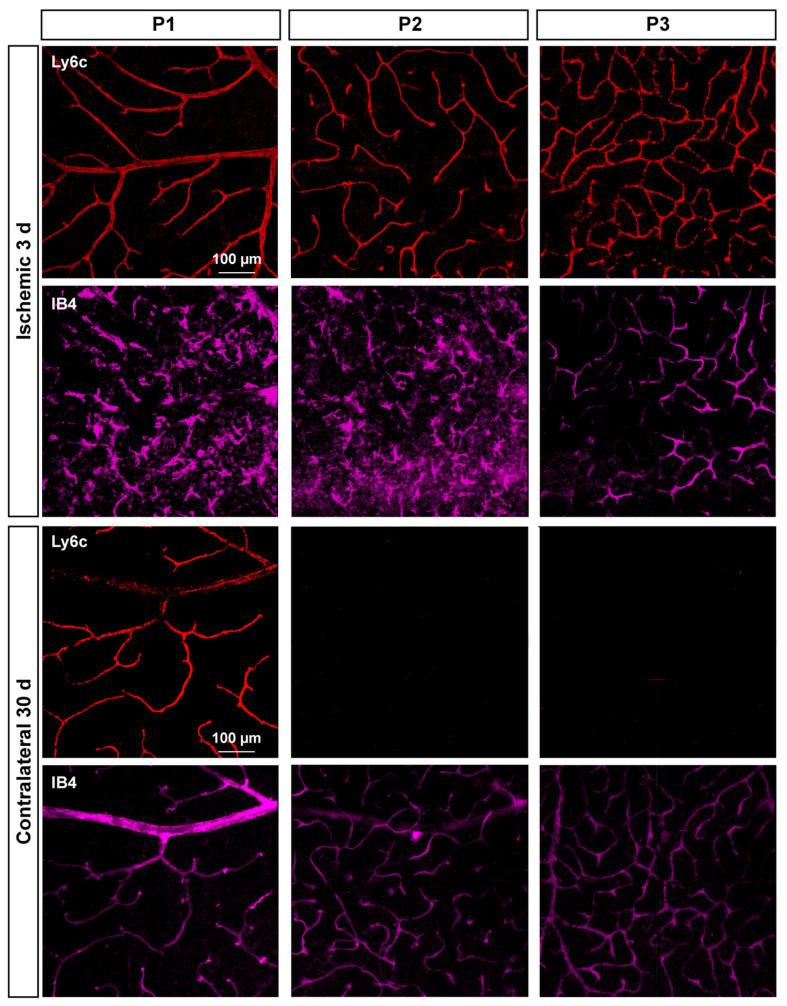
Microglial activation in the injured retinas and down-regulation of Ly6c in the contralateral ones. Confocal images showing Ly6c and IB4 staining in the three vascular plexuses of injured and contralateral retinas analyzed at 3 and 30 days after the induction of the ischemia, respectively.

In P2 and P3, there was a gradual loss of Ly6c expression (Figure 8) until it became almost undetectable at 30 days. To elucidate whether this reflected a loss of P2 and P3 vessels or a down-regulation of Ly6c expression, a double staining with IB4 was performed. As the positive control we used injured retinas (Figure 9). In the injured retinas, Ly6c marked the three vascular plexuses, while IB4 staining revealed abundant activated microglial cells in P1 and P2 around the vessels, masking them. In P3, the IB4 and Ly6c signal was circumscribed to the vessels. In the contralateral retinas 30 days after the ischemic insult, Ly6c expression in P2 and P3 had disappeared but not the vascular plexuses, which were clearly observed with IB4. These results indicate that the loss of the Ly6c signal in the contralateral P2 and P3 plexuses was due to a down-regulation of Ly6c rather that to a regression of the vasculature.

## 3. Discussion

As we see here, Ly6c, a marker of classically activated monocyte/macrophages [33,34], is also a good marker to identify blood vessels in mice.

We have focused this work on the retina, but we also show that Ly6c detects vessels in the brain, in accordance with previous studies [30] in the heart, intestine, kidney, and tail. Therefore, Ly6c is expressed in the vasculature of tissues derived from the three germinal layers, and in continuous and fenestrated capillaries [40,41].

In the retina, Ly6c immunodetection presents two main advantages over the traditional staining with IB4 isolectin [11,12,13,14]. Firstly, the Ly6c signal is similar in arteries, veins, and capillaries, allowing the study of all the components of the vascular system. IB4 binds to the α-gal residues present on the blood vessels, but because veins have a lower density of them than arterioles, IB4 staining in veins is weak [42]. Secondly, Ly6c is more selective of blood vessels than IB4. IB4 also binds to activated microglial cells as seen here and elsewhere [43], a binding that impairs the analysis of the vasculature in pathological situations. Certainly, as shown here, Ly6c also detects classically activated monocyte/macrophages (Ly6c^high^) infiltrated in the retinal parenchyma. This is an advantage because it allows for assessment of whether a given insult to the CNS affects the blood–brain/retinal barrier permeability.

Using our experimental conditions, the Ly6c signal was brighter than that of IB4, and so imaging fine peripheral capillaries with IB4 was difficult, in contrast to other reports [44]. The choice of fluorochrome (555 for Ly6c and 647 for IB4) may have had an impact because the 647 signal is weaker. However, in Figure 1B, Ly6c was detected with a 647 fluorophore and its signal was still brighter than that of IB4. Thus, these differences, we believe, may be due to the different protocols: while we used IB4 directly coupled to fluorescence, Pitulescu et al. [44] used biotinylated IB4 which was subsequently identified with avidin-fluorophore, hence amplifying the signal.

The retinal superficial vascular plexus is formed by veins (6) and arteries (6) that later branch into venules and arterioles to finally give rise to capillaries. In the last stages of P1 development, some vessels enter the retina originating the deep plexus (P3), which is mostly made up of capillaries with a saccular morphology. Finally, a network of capillaries is established between plexuses one and three, giving rise to the intermediate plexus (P2) [4,45]. Ly6c immunodetection allows the three plexuses to be imaged and the fraction of the retinal area that they occupy to be quantified, with results similar to previous reports using other markers [16].

Most works on glaucoma models are focused on the inner layers of the injured retina with the main goals of deciphering the underlying causes of the anatomical and functional loss of RGCs and testing neuroprotective therapies [46,47]. Previous works from our group [35,37,48] report the functional and anatomical changes following AOHT, in both the inner and outer retina. Here we continue those studies with the analyses of the vasculature in all retinal layers and in both retinas.

In the injured retinas, P2 and P3 could not be measured because the outer retina thinned, and they became indistinguishable. In P1, there was a loss of Ly6c expression around the optic nerve head right after the reperfusion and up to 3 days. Retinas processed immediately after were very fragile and difficult to dissect. However, the tissue recovered at 24 h. At these early time points, central arteries/veins appeared ruptured, with a discontinuous Ly6C expression, and there was extravasation of monocyte/macrophages. Vessel rupture and brain–blood barrier impairment is a common occurrence in stroke and ischemia/reperfusion injuries [48], which in our model may be worsened by the acute rise of the intraocular pressure up to 10 times physiological values.

At 15 days, extra-numerary twisted capillaries were observed, and this vascular growth reached significance at 30 days. These results are in agreement with previous studies showing that a decrease in oxygen supply can lead to vascular tortuousness and neovascularization to meet metabolic demands [21,49].

The contralateral effect is a puzzling phenomenon of yet unknown etiology, where the uninjured tissue/organ of a bilateral system responds to the injury performed in the other one (reviewed in [50]). Most current works on the contralateral response are described in the visual system. This effect has been observed in different models of unilateral retinal injuries, and comprises several changes, from RGC death to microglial and macroglial activation [51,52,53,54,55,56,57,58,59].

Contrary to that reported in most studies in which the contralateral effect is an attenuated and delayed reflection of what occurs in the damaged tissue [51,52,53,54,55,56,57,58,59,60,61], we see here a different contralateral response: while in the injured retina, the area of the superficial plexus increased in the contralateral one decreased with time slowly but significantly. Furthermore, the intermediate and deep plexuses of the contralateral retina down-regulated Ly6c expression, an effect that was not observed in the injured one.

What is the meaning of this behavior? At this stage we do not know, and further experiments are needed to elucidate this point. However, we poise the hypothesis that Ly6c plays an important role in vasculature recovery and/or monocyte/macrophage homing, and therefore its expression must be reduced in the contralateral eye to meet the needs of the injured one.

In conclusion, the main finding of this work is that Ly6c immunodetection is a new and precise approach to detect and quantify retinal vascular plexuses in health and disease, and to assess monocyte/macrophage infiltration after damage.

## 4. Materials and Methods

### 4.1. Animal Handling

Animal procedures conformed to the ARVO Statement for the Use of Animals in Ophthalmic and Vision Research and adhered to the ARRIVE guidelines. Adult pigmented C57BL/6 mice were obtained from the University of Murcia breeding colony. All animals were treated in compliance with the European Union guidelines for Animal Care and Use for Scientific Purpose (Directive 2010/63/EU) and the guidelines from the Association for Research in Vision and Ophthalmology (ARVO) Statement for the Use of Animals in Ophthalmic and Vision Research. All procedures were approved by the Ethical and Animal Studies Committee of the University of Murcia, Spain (approved protocols: A1320140704, A13170110, and A13170111).

### 4.2. Acute Ocular Hypertension (AOHT) Induction

AOHT was induced to the experimental group as described [36,37]. Briefly, anesthetized mice were placed over a heating pad to maintain normal body temperature. A 30-gauge infusion needle placed in the anterior chamber of the left eye was connected to a 500 mL container of 0.9% NaCl 1.2 m above the eye. Intra-ocular pressure (IOP) was raised from baseline (8 ± 2 mmHg) to 87 ± 4 mmHg, as monitored with a Tono-Pen (Tono-Pen; Medtronic Co., Dublin, Ireland) [37,51,60]. Following 90 min, the needle was removed and the IOP returned to basal values. Retinal blood flow was examined by direct fundoscopy with an operating microscope (Spot OPMI 11, Carl Zeiss, Oberkochen, Germany) prior to, during, and after acute OHT. While AOHT resulted in lack of retinal perfusion, there was complete blood flow reperfusion after the removal of the needle. During and after the procedure, the corneas were covered with an ointment (Tobrex; Alcon S. A., Barcelona, Spain) to prevent desiccation.

Both retinas were analyzed at 0, 24 h, 3, 15, or 30 days after the induction of the AOHT (n = 4/time point). Retinas from intact animals were used as controls (n = 8). No exclusion criteria were enforced, and all animals were considered. In total, 28 mice were used in the present study.

### 4.3. Tissue Processing

Animals were sacrificed with an intraperitoneal injection of an overdose of sodium pentobarbital (Dolethal, Vetoquinol; Especialidades Veterinarias, S.A., Alcobendas, Madrid, Spain). and perfused transcardially with saline and 4% paraformaldehyde (PFA) in 0.1 M phosphate buffer.

The eyes were enucleated and fixed for one extra hour in 4% PFA at room temperature. Both retinas were dissected and prepared as flattened whole-mounts maintaining the retinal orientation by making four radial cuts [52]. Flat mounts were maintained for 1 h at RT in PFA 4% and then kept in PBS until immunodetection, which is the recommended fixation for IB4 detection [44].

The brain, kidney, intestine, heart, and tail from some intact animals were dissected and post-fixed in 4% PFA at 4 °C overnight, and then cryoprotected in increasing concentrations of sucrose before embedding them in an optimal cutting temperature (OCT) compound (Sakura Finetek, Torrance, CA, USA). Serial sections (15–30 μm thick) were obtained on a cryostat.

To analyze the non-perfused retinas, eyes were enucleated and fixated in 4% PFA at 4 °C overnight. Thereafter, flat mounts were processed as above.

### 4.4. Immunodetection

Whole-mounted and cross-sections retinas were immunodetected as previously described [35,36,37,43,52,59,60,61,62,63] with rat α-Ly-6C primary antibody (1:500; sc-52650, Santa Cruz Biotechnology Inc., Dallas, TX, USA) and mouse monoclonal α-CD115 (1:200; sc-46662, Santa Cruz Biotechnology Inc., Dallas, TX, USA). Secondary detection was carried out with goat α-rat Alexa 555 or 647 and goat α-mouse IgG1 Alexa 488 secondary antibodies diluted 1:500 (Moleculasr Probes; Thermo Fisher Scientific, Madrid, Spain. Cat. Number A21434, A21247, A21121). For comparative purposes, the isolectin GS-IB4 from *Griffonia simplicifolia* coupled to Alexa 647 (1:300, Molecular Probes, Thermo Fisher Scientific Alcobendas, Madrid, Spain) was used in combination with Ly6C immunodetection. No signal was observed when testing the cross-reactivity of the secondary antibodies in samples without the primary antibody.

In brief, after permeabilization, samples were incubated overnight at 4 °C with the primary antibody diluted in blocking buffer (phosphate buffer saline (PBS) with 2% donkey normal serum and 2% Triton for flat mounts and 0.2% for cross sections). Then, they were washed three times in PBS and incubated for 2 h at room temperature with the secondary antibody and IB4 when required. Finally, samples were thoroughly washed in PBS and mounted in slides covered with antifading solution (Vectashield, Vector laboratories, Palex Medical, Barcelona, Spain). In all sections, nuclei were counterstained with DAPI (Vectashield with DAPI).

### 4.5. Image Acquisition and Analysis

Images were acquired using a Leica DM6B epifluorescence microscope or a Leica SP8 confocal microscope (Leica Microsystems, Wetzlar, Germany). Retinal and brain photomontages were reconstructed from 64 (11 × 14) individual images. Photomontages of the three vascular plexuses were acquired from the same retina, focusing on the Ly6c signal in the corresponding retinal layer, and the color acquisition settings were red (P1), purple (P2), and green (P3). The area fraction of Ly6c staining was measured by ImageJ software (developed by Wayne Rasband, National Institutes of Health, Bethesda, MD, USA; https://imagej.nih.gov/ij (15 May 2021) and defined as the number of pixels which form the retinal vessels over the total area of the retina. Ischemia versus control measures were blinded.

### 4.6. Statistics

Data were analyzed and plotted with GraphPad Prism v.7 (GraphPad, San Diego, CA, USA). Anatomical data are presented as mean ± standard deviation (SD). Differences were considered significant when *p* < 0.05. The tests are detailed in the results.

## Figures and Tables

**Table 1:** Markers to identify mouse blood vessels.

Marker	Target	Characteristics	References
Isolectin-IB4	Terminal α-d-galactosyl residues	Binds to blood vessels and to activated microglial cells	[5,11,12,13,14,15,16]
CD31 (cluster of differentiation 31) also known as PECAM-1 (platelet endothelial cell adhesion molecule)	Adhesion molecule that constitutes a large part of the intercellular junctions of endothelial cells	It is found on endothelial cells, platelets, Kupffer cells, macrophages, granulocytes, lymphocytes, megakaryocytes, osteoclasts, and in certain tumors	[12,16,17,18]
ICAM2 (intercellular adhesion molecule 2) also known as CD102 (cluster of differentiation 102)	Type I transmembrane glycoprotein present in the apical/luminal endothelial cell membrane	ICAM2 masks highlight vessel segments undergoing remodeling. It mediates adhesive interactions important for antigen-specific immune response	[11,18,19]
CLDN5 (claudin 5)	It is one of the six high abundant tight junction proteins in the blood–brain barrier in vivo and the dominant one in vitro	Transiently expressed in the retinal pigment epithelium (RPE) during development, where its expression correlates with permeability changes in the developing RPE	[12,20]
ColIV or Col4 (collagen IV)	One of the main components of the basement membrane (BM), a specialized extracellular matrix that compartmentalizes tissues, provides structural support, and influences cell behavior and signaling	Collagen IV is the most abundant structural BM component and is essential for BM integrity but not initial BM formation	[11,19,21]
Endoglin (ENG) also known as CD105	Transmembrane glycoprotein that functions as a coreceptor for ligands of the transforming growth factor-β superfamily. It is predominantly expressed by activated endothelial cells	It is a facilitator of ligand binding and has a crucial role in angiogenesis. It is also a marker of mesenchymal stem cells and it is expressed in progenitor cells involved in vascular remodeling in animal models	[22,23,24]
ZO-1 (zonula occludens-1) also known as TJP1 (tight junction protein-1)	One of the proteins that create intercellular boundaries between the plasma membrane domains of epithelial and endothelial cells (endothelial cell–cell junctions)	Is thought to have both structural and signaling roles. It can also associate with claudin, occludin, and F-actin, at tight junction stands, where it provides a linkage between the actin cytoskeleton and the tight junction	[11,16,25]
CDH5 (cadherin 5) also known as VE-cadherin	It is a strictly endothelial specific adhesion molecule located at junctions between endothelial cells and promotes homotypic cell-to-cell interaction	It is vital for the maintenance and control of endothelial cell contacts. It is relevant for the control of vascular permeability and leukocyte extravasation and regulates various cellular processes such as cell proliferation and apoptosis and modulates vascular endothelial growth factor receptor functions. It is essential during embryonic angiogenesis	[11,16]
Erg (ETS-related gene)	It is expressed in the nuclei of endothelial cells	It is a transcription factor that has been linked to angiogenesis and to the promotion of vascular stability	[11,26]
Dextran-fluorophore (complex branched glucan labelled with a fluorophore)	Blood vessel lumen	Used for intravascular perfusion. High-molecular-weight FITC-dextran remains in the vasculature without diffusion	[27,28]
gel-BSA-FITC (gel-bovine serum albumin-fluorescein isothiocyanate)	Blood vessel lumen	Used for intravascular perfusion. The high molecular weight of albumin prevents the marker from crossing blood vessel walls, which ensures the confinement of the fluorescent signal within the blood vessels	[29]

## Data Availability

The data presented in this study are available on request from the corresponding authors.

## Abbreviations

Ly6c	Lymphocyte antigen 6 complex
CNS	Central nervous system
RGC	Retinal ganglion cells
CD31	Cluster of differentiation 31
ICAM2	Intercellular adhesion molecule 2
CLDN5	Claudin 5
Col4	Collagen 4
ENG	Endoglin
ZO-1	Zonula occludens-1
CDH5	Cadherin 5
Erg	ETS-related gene
BSA	Bovine serum albumin
P (1–3)	Vascular plexus
IOP	Intraocular pressure
OHT	Ocular hypertension
AOHT	Acute ocular hypertension
PFA	Paraformaldehyde
OCT	Optimal cutting temperature compound
PBS	Phosphate buffered saline
SD	Standard deviation

## References

[1] Lendahl U., Nilsson P., Betsholtz C. (2019). Emerging Links between Cerebrovascular and Neurodegenerative Diseases-a Special Role for Pericytes. EMBO Rep..

[2] Sweeney M.D., Kisler K., Montagne A., Toga A.W., Zlokovic B.V. (2018). The Role of Brain Vasculature in Neurodegenerative Disorders. Nat. Neurosci..

[3] Cortes-Canteli M., Iadecola C. (2020). Alzheimer’s Disease and Vascular Aging: JACC Focus Seminar. J. Am. Coll. Cardiol..

[4] Sun Y., Smith L.E.H. (2018). Retinal Vasculature in Development and Diseases. Annu. Rev. Vis. Sci..

[5] Almasieh M., MacIntyre J.N., Pouliot M., Casanova C., Vaucher E., Kelly M.E.M., Di Polo A. (2013). Acetylcholinesterase Inhibition Promotes Retinal Vasoprotection and Increases Ocular Blood Flow in Experimental Glaucoma. Investig. Ophthalmol. Vis. Sci..

[6] Colucciello M. (2005). Retinal Vascular Disease in Hypertension. Postgrad. Med..

[7] Schuster A.K., Erb C., Hoffmann E.M., Dietlein T., Pfeiffer N. (2020). The Diagnosis and Treatment of Glaucoma. Dtsch. Arztebl. Int..

[8] Kimura A., Namekata K., Guo X., Noro T., Harada C., Harada T. (2017). Targeting Oxidative Stress for Treatment of Glaucoma and Optic Neuritis. Oxid. Med. Cell. Longev..

[9] Harder J.M., Williams P.A., Braine C.E., Yang H.S., Thomas J.M., Foxworth N.E., John S.W.M., Howell G.R. (2020). Complement Peptide C3a Receptor 1 Promotes Optic Nerve Degeneration in DBA/2J Mice. J. Neuroinflamm..

[10] He Z., Vingrys A.J., Armitage J.A., Bui B.V. (2011). The Role of Blood Pressure in Glaucoma. Clin. Exp. Optom..

[11] Franco C.A., Jones M.L., Bernabeu M.O., Geudens I., Mathivet T., Rosa A., Lopes F.M., Lima A.P., Ragab A., Collins R.T. (2015). Dynamic Endothelial Cell Rearrangements Drive Developmental Vessel Regression. PLoS Biol..

[12] Rust R., Grönnert L., Dogançay B., Schwab M.E. (2019). A Revised View on Growth and Remodeling in the Retinal Vasculature. Sci. Rep..

[13] Fu Z., Löfqvist C.A., Liegl R., Wang Z., Sun Y., Gong Y., Liu C., Meng S.S., Burnim S.B., Arellano I. (2018). Photoreceptor Glucose Metabolism Determines Normal Retinal Vascular Growth. EMBO Mol. Med..

[14] Dudiki T., Meller J., Mahajan G., Liu H., Zhevlakova I., Stefl S., Witherow C., Podrez E., Kothapalli C.R., Byzova T.V. (2020). Microglia Control Vascular Architecture via a TGFβ1 Dependent Paracrine Mechanism Linked to Tissue Mechanics. Nat. Commun..

[15] Shen W., Fruttiger M., Zhu L., Chung S.H., Barnett N.L., Kirk J.K., Lee S.R., Coorey N.J., Killingsworth M., Sherman L.S. (2012). Conditional Müller Cell Ablation Causes Independent Neuronal and Vascular Pathologies in a Novel Transgenic Model. J. Neurosci..

[16] Franco C.A., Blanc J., Parlakian A., Blanco R., Aspalter I.M., Kazakova N., Diguet N., Mylonas E., Gao-Li J., Vaahtokari A. (2013). SRF Selectively Controls Tip Cell Invasive Behavior in Angiogenesis. Development.

[17] Kim S.J., Kim S.A., Choi Y.A., Park D.Y., Lee J. (2020). Alpha-Smooth Muscle Actin-Positive Perivascular Cells in Diabetic Retina and Choroid. Int. J. Mol. Sci..

[18] Halai K., Whiteford J., Ma B., Nourshargh S., Woodfin A. (2014). ICAM-2 Facilitates Luminal Interactions between Neutrophils and Endothelial Cells in Vivo. J. Cell Sci..

[19] Zhou Q., Perovic T., Fechner I., Edgar L.T., Hoskins P.R., Gerhardt H., Krüger T., Bernabeu M.O. (2021). Association between Erythrocyte Dynamics and Vessel Remodelling in Developmental Vascular Networks. J. R. Soc. Interface.

[20] Berndt P., Winkler L., Cording J., Breitkreuz-Korff O., Rex A., Dithmer S., Rausch V., Blasig R., Richter M., Sporbert A. (2019). Tight Junction Proteins at the Blood–Brain Barrier: Far More than Claudin-5. Cell. Mol. Life Sci..

[21] Scott A., Powner M.B., Fruttiger M. (2014). Quantification of Vascular Tortuosity as an Early Outcome Measure in Oxygen Induced Retinopathy (OIR). Exp. Eye Res..

[22] Tual-chalot S., Mahmoud M., Allinson K.R., Redgrave R.E., Zhai Z., Oh S.P., Fruttiger M., Arthur H.M. (2014). Endothelial Depletion of Acvrl1 in Mice Leads to Arteriovenous Malformations Associated with Reduced Endoglin Expression. PLoS ONE.

[23] Rabelink T.J., Lebrin F. (2021). Thresholds of Endoglin Expression in Endothelial Cells Explains Vascular Etiology in Hereditary Hemorrhagic Telangiectasia Type 1. Int. J. Mol. Sci..

[24] Barnett J.M., Suarez S., McCollum G.W., Penn J.S. (2014). Endoglin Promotes Angiogenesis in Cell- and Animal-Based Models of Retinal Neovascularization. Investig. Ophthalmol. Vis. Sci..

[25] Tornavaca O., Chia M., Dufton N., Almagro L.O., Conway D.E., Randi A.M., Schwartz M.A., Matter K., Balda M.S. (2015). ZO-1 Controls Endothelial Adherens Junctions, Cell–Cell Tension, Angiogenesis, and Barrier Formation. J. Cell Biol..

[26] Shah A.V., Birdsey G.M., Peghaire C., Pitulescu M.E., Dufton N.P., Yang Y., Weinberg I., Osuna Almagro L., Payne L., Mason J.C. (2017). The Endothelial Transcription Factor ERG Mediates Angiopoietin-1-Dependent Control of Notch Signalling and Vascular Stability. Nat. Commun..

[27] Li S., Li T., Luo Y., Yu H., Sun Y., Zhou H., Liang X., Huang J., Tang S. (2011). Retro-Orbital Injection of FITC-Dextran Is an Effective and Economical Method for Observing Mouse Retinal Vessels. Mol. Vis..

[28] Auffray C., Fogg D., Garfa M., Elain G., Join-Lambert O., Kayal S., Sarnacki S., Cumano A., Lauvau G., Geissmann F. (2007). Monitoring of Blood Vessels and Tissues by a Population of Monocytes with Patrolling Behavior. Science.

[29] Di Giovanna A.P., Tibo A., Silvestri L., Müllenbroich M.C., Costantini I., Allegra Mascaro A.L., Sacconi L., Frasconi P., Pavone F.S. (2018). Whole-Brain Vasculature Reconstruction at the Single Capillary Level. Sci. Rep..

[30] Alliot F., Rutin J., Pessac B. (1998). Ly-6C Is Expressed in Brain Vessels Endothelial Cells but Not in Microgila of the Mouse. Neurosci. Lett..

[31] Trost A., Motloch K., Bruckner D., Schroedl F., Bogner B., Kaser-Eichberger A., Runge C., Strohmaier C., Klein B., Aigner L. (2015). Time-Dependent Retinal Ganglion Cell Loss, Microglial Activation and Blood-Retina-Barrier Tightness in an Acute Model of Ocular Hypertension. Exp. Eye Res..

[32] Duijvestijn A.M., van Goor H., Klatter F., Majoor G.D., van Bussel E., van Breda Vriesman P.J. (1992). Antibodies Defining Rat Endothelial Cells: RECA-1, a Pan-Endothelial Cell-Specific Monoclonal Antibody. Lab. Investig..

[33] Alkhani A., Levy C.S., Tsui M., Rosenberg K.A., Polovina K., Mattis A.N., Mack M., Van Dyken S., Wang B.M., Maher J.J. (2020). Ly6cLo Non-Classical Monocytes Promote Resolution of Rhesus Rotavirus-Mediated Perinatal Hepatic Inflammation. Sci. Rep..

[34] Teh Y.C., Ding J.L., Ng L.G., Chong S.Z. (2019). Capturing the Fantastic Voyage of Monocytes Through Time and Space. Front. Immunol..

[35] Rovere G., Nadal-Nicolás F.M., Wang J., Bernal-Garro J.M., García-Carrillo N., Villegas-Pérez M.P., Agudo-Barriuso M., Vidal-Sanz M. (2016). Melanopsin-Containing or Non-Melanopsin–Containing Retinal Ganglion Cells Response to Acute Ocular Hypertension With or Without Brain-Derived Neurotrophic Factor Neuroprotection. Investig. Ophthalmol. Vis. Sci..

[36] Wang J., Valiente-Soriano F.J., Nadal-Nicolás F.M., Rovere G., Chen S., Huang W., Agudo-Barriuso M., Jonas J.B., Vidal-Sanz M., Zhang X. (2017). MicroRNA Regulation in an Animal Model of Acute Ocular Hypertension. Acta Ophthalmol..

[37] Gallego-Ortega A., Norte-Muñoz M., Miralles de Imperial-Ollero J.A., Bernal-Garro J.M., Valiente-Soriano F.J., de la Villa Polo P., Avilés-Trigueros M., Villegas-Pérez M.P., Vidal-Sanz M. (2020). Functional and Morphological Alterations in a Glaucoma Model of Acute Ocular Hypertension. Prog. Brain Res..

[38] Honda M., Surewaard B.G.J., Watanabe M., Hedrick C.C., Lee W.-Y., Brown K., McCoy K.D., Kubes P. (2020). Perivascular Localization of Macrophages in the Intestinal Mucosa Is Regulated by Nr4a1 and the Microbiome. Nat. Commun..

[39] Sherr C.J., Roussel M.F., Rettenmier C.W. (1988). Colony-Stimulating Factor-1 Receptor (c-Fms). J. Cell. Biochem..

[40] Hallmann R., Horn N., Selg M., Wendler O., Pausch F., Sorokin L.M. (2005). Expression and Function of Laminins in the Embryonic and Mature Vasculature. Physiol. Rev..

[41] Malik A.B., Lynch J.J., Cooper J.A. (1989). Endothelial Barrier Function. J. Investig. Dermatol..

[42] Hansen-Smith F.M., Watson L., Lu D.Y., Goldstein I. (1988). Griffonia Simplicifolia I: Fluorescent Tracer for Microcirculatory Vessels in Nonperfused Thin Muscles and Sectioned Muscle. Microvasc. Res..

[43] Di Pierdomenico J., García-Ayuso D., Pinilla I., Cuenca N., Vidal-Sanz M., Agudo-Barriuso M., Villegas-Pérez M.P. (2017). Early Events in Retinal Degeneration Caused by Rhodopsin Mutation or Pigment Epithelium Malfunction: Differences and Similarities. Front. Neuroanat..

[44] Pitulescu M.E., Schmidt I., Benedito R., Adams R.H. (2010). Inducible Gene Targeting in the Neonatal Vasculature and Analysis of Retinal Angiogenesis in Mice. Nat. Protoc..

[45] Selvam S., Kumar T., Fruttiger M. (2018). Retinal Vasculature Development in Health and Disease. Prog. Retin. Eye Res..

[46] Romano G.L., Amato R., Lazzara F., Porciatti V., Chou T.-H., Drago F., Bucolo C. (2020). P2X7 Receptor Antagonism Preserves Retinal Ganglion Cells in Glaucomatous Mice. Biochem. Pharmacol..

[47] Vidal-Sanz M., Valiente-Soriano F.J., Ortín-Martínez A., Nadal-Nicolás F.M., Jiménez-López M., Salinas-Navarro M., Alarcón-Martínez L., García-Ayuso D., Avilés-Trigueros M., Agudo-Barriuso M. (2015). Retinal Neurodegeneration in Experimental Glaucoma. Prog. Brain Res..

[48] Sarvari S., Moakedi F., Hone E., Simpkins J.W., Ren X. (2020). Mechanisms in Blood-Brain Barrier Opening and Metabolism-Challenged Cerebrovascular Ischemia with Emphasis on Ischemic Stroke. Metab. Brain Dis..

[49] Gariano R.F., Gardner T.W. (2005). Retinal Angiogenesis in Development and Disease. Nature.

[50] Lucas-Ruiz F., Galindo-Romero C., Albaladejo-García V., Vidal-Sanz M., Agudo-Barriuso M. (2021). Mechanisms Implicated in the Contralateral Effect in the Central Nervous System after Unilateral Injury: Focus on the Visual System. Neural Regen. Res..

[51] Valiente-Soriano F.J., Salinas-Navarro M., Jiménez-López M., Alarcón-Martínez L., Ortín-Martínez A., Bernal-Garro J.M., Avilés-Trigueros M., Agudo-Barriuso M., Villegas-Pérez M.P., Vidal-Sanz M. (2015). Effects of Ocular Hypertension in the Visual System of Pigmented Mice. PLoS ONE.

[52] Dekeyster E., Aerts J., Valiente-Soriano F.J., De Groef L., Vreysen S., Salinas-Navarro M., Vidal-Sanz M., Arckens L., Moons L. (2015). Ocular Hypertension Results in Retinotopic Alterations in the Visual Cortex of Adult Mice. Curr. Eye Res..

[53] Lucas-Ruiz F., Galindo-Romero C., Rodríguez-Ramírez K.T., Vidal-Sanz M., Agudo-Barriuso M. (2019). Neuronal Death in the Contralateral Un-Injured Retina after Unilateral Axotomy: Role of Microglial Cells. Int. J. Mol. Sci..

[54] Ramírez A.I., Fernández-Albarral J.A., de Hoz R., López-Cuenca I., Salobrar-García E., Rojas P., Valiente-Soriano F.J., Avilés-Trigueros M., Villegas-Pérez M.P., Vidal-Sanz M. (2020). Microglial Changes in the Early Aging Stage in a Healthy Retina and an Experimental Glaucoma Model. Prog. Brain Res..

[55] Fernández-Albarral J.A., Salazar J.J., de Hoz R., Marco E.M., Martín-Sánchez B., Flores-Salguero E., Salobrar-García E., López-Cuenca I., Barrios-Sabador V., Avilés-Trigueros M. (2021). Retinal Molecular Changes Are Associated with Neuroinflammation and Loss of RGCs in an Experimental Model of Glaucoma. Int. J. Mol. Sci..

[56] Di Pierdomenico J., García-Ayuso D., Jiménez-López M., Agudo-Barriuso M., Vidal-Sanz M., Villegas-Pérez M.P. (2016). Different Ipsi- and Contralateral Glial Responses to Anti-VEGF and Triamcinolone Intravitreal Injections in Rats. Investig. Ophthalmol. Vis. Sci..

[57] González-Riquelme M.J., Galindo-Romero C., Lucas-Ruiz F., Martínez-Carmona M., Rodríguez-Ramírez K.T., Cabrera-Maqueda J.M., Norte-Muñoz M., Vidal-Sanz M., Agudo-Barriuso M. (2021). Axonal Injuries Cast Long Shadows: Long Term Glial Activation in Injured and Contralateral Retinas after Unilateral Axotomy. Int. J. Mol. Sci..

[58] Sánchez-Migallón M.C., Valiente-Soriano F.J., Salinas-Navarro M., Nadal-Nicolás F.M., Jiménez-López M., Vidal-Sanz M., Agudo-Barriuso M. (2018). Nerve Fibre Layer Degeneration and Retinal Ganglion Cell Loss Long Term after Optic Nerve Crush or Transection in Adult Mice. Exp. Eye Res..

[59] Galindo-Romero C., Valiente-Soriano F.J., Jiménez-López M., García-Ayuso D., Villegas-Pérez M.P., Vidal-Sanz M., Agudo-Barriuso M. (2013). Effect of Brain-Derived Neurotrophic Factor on Mouse Axotomized Retinal Ganglion Cells and Phagocytic Microglia. Investig. Ophthalmol. Vis. Sci..

[60] Valiente-Soriano F.J., Nadal-Nicolás F.M., Salinas-Navarro M., Jiménez-López M., Bernal-Garro J.M., Villegas-Pérez M.P., Agudo-Barriuso M., Vidal-Sanz M. (2015). BDNF Rescues RGCs But Not Intrinsically Photosensitive RGCs in Ocular Hypertensive Albino Rat Retinas. Investig. Ophthalmol. Vis. Sci..

[61] Shenker N., Haigh R., Roberts E., Mapp P., Harris N., Blake D. (2003). A Review of Contralateral Responses to a Unilateral Inflammatory Lesion. Rheumatology.

[62] Ramírez A.I., Salazar J.J., de Hoz R., Rojas B., Gallego B.I., Salinas-Navarro M., Alarcón-Martínez L., Ortín-Martínez A., Avilés-Trigueros M., Vidal-Sanz M. (2010). Quantification of the Effect of Different Levels of IOP in the Astroglia of the Rat Retina Ipsilateral and Contralateral to Experimental Glaucoma. Investig. Ophthalmol. Vis. Sci..

[63] De Hoz R., Gallego B.I., Ramírez A.I., Rojas B., Salazar J.J., Valiente-Soriano F.J., Avilés-Trigueros M., Villegas-Perez M.P., Vidal-Sanz M., Triviño A. (2013). Rod-like Microglia Are Restricted to Eyes with Laser-Induced Ocular Hypertension but Absent from the Microglial Changes in the Contralateral Untreated Eye. PLoS ONE.

